# A Bioinformatics and Wet-Lab-Based Pipeline Identifies *CLDN10* and *GJB2* as Epigenetically Silenced Tumor Suppressor Genes in Cutaneous Melanoma

**DOI:** 10.3390/ijms27052483

**Published:** 2026-03-08

**Authors:** Sarah Arroyo Villora, Veit Xaver Baumann, Yufen Zhao, Niklas Philipp, Reinhard H. Dammann, Cornelia Sigges, Antje Maria Richter

**Affiliations:** 1 Institute for Genetics, Justus Liebig University Giessen, 35390 Giessen, Germany; 2 Department of Mathematics, Natural Sciences and Computer Science, University of Applied Sciences Mittelhessen, 35390 Giessen, Germany

**Keywords:** DNA (hyper)methylation, tumor suppressor gene, epigenetics, methylome, transcriptome, omics, database, skin cutaneous melanoma, *CLDN10*, *GJB2*

## Abstract

Studying epigenetic changes in cancer development can reveal the role of tumor suppressor genes and their regulation by DNA methylation. CpG islands, found in promoter regions, are of particular interest, as their hypermethylation can silence tumor suppressor gene expression. Here, we present a practical analysis pipeline for wet-lab biologists with the aim of identify novel epigenetically regulated tumor suppressors using freely available online tools. Bioinformatic platforms such as the R2 Genomics Analysis and Visualization Platform enable analysis of genomic organization, CpG islands, and regulatory elements. Differential methylation and gene expression analyses are based on datasets including TCGA, using tools such as MethSurv, TCGA Wanderer, and GEPIA2 to correlate DNA methylation with gene expression. This bioinformatic step is the basis for the tumor suppressor verification in the wet-lab. Using this pipeline, we identified *CLDN10* and *GJB2* as potential tumor suppressors in melanoma. Experimentally, our approach includes DNA methylation analysis based on DNA bisulfite conversion, combined bisulfite restriction analysis (CoBRA), pyrosequencing for specific CpG methylation quantification, and RT-PCR for RNA expression quantification. We verify these results in primary tumors, metastases, and cell line models. This approach supports efficient identification of novel epigenetically regulated tumor suppressors, providing practical research guidelines.

## 1. Introduction

Cancer development can often be traced back to alterations in two specific gene classes: oncogenes and tumor suppressor genes. Proto-oncogenes promote cell growth and proliferation, whereas tumor suppressor genes (TSGs) prevent uncontrolled proliferation [1,2]. The two gene classes usually balance cell growth. In cancer, however, this balance is lost by the gain of function of the so-called oncogenes and by the loss of function of TSGs. The result is uncontrolled cell growth and the promotion of tumor development [1].

This phenomenon of loss of function of tumor suppressors is often associated with their epigenetic regulation [3,4,5,6,7,8]. Genes can exhibit abnormal expression through epigenetic activation or inactivation and be a causal factor in cancer [9]. Fundamental epigenetic mechanisms include histone modifications and DNA methylation, which regulate gene expression [10]. Transcriptional repression can be induced, for example, by the Polycomb Repressive Complex 2 (PRC2) and its catalytic subunit Enhancer of zeste homolog 2 (EZH2) [11]. DNA methylation occurs via DNA methyltransferases (DNMTs) and their addition of a methyl group at the C-5 position of a cytosine in the context of a subsequent guanine (CpG dinucleotide) [12,13]. The result is the formation of 5-methylcytosine (5 mC). CpG dinucleotides have become rare in the human genome by evolutionary selection. However, CpG dinucleotides are enriched in promoter-associated CpG islands (CGIs), present in over half of vertebrate genes and close to transcription start sites (TSSs) [9,14,15,16]. DNA methylation in promoter regions can silence the expression of the associated gene or gene isoform linked to the very CGI by the formation of gene silencing complexes [17]. When identifying these epigenetically silenced TSGs, it is crucial to note that hypermethylation of the gene is highly tumor entity-dependent.

In this study, malignant melanoma is used as the example tumor type in the pipeline for identifying tumor suppressors. Malignant melanoma is the deadliest form of skin cancer [18]. We use various available bioinformatic online tools for the efficient identification of such TSGs and present a pipeline for wet-lab biologists. Promising candidate genes from TCGA datasets can show progressive hypermethylation and reduced expression [19,20]. Here, we identified the two genes *CLDN10* and *GJB2* as potential TSGs in malignant melanoma. We selected *CLDN10* and *GJB2* based on prior functional studies, where CLDN10 was investigated in the context of kidney disease and GJB2 in wound healing. This background made them compelling candidates. In our previous studies, we confirmed CLDN10 as an epigenetically silenced tumor suppressor in renal cancer, including post-transplant clear cell renal carcinoma (PT-ccRCC) [21]. Furthermore, loss of CLDN10 has been linked to metastatic melanoma and interleukin deficiency, although without isoform-specific distinction [22]. Cx26 may also contribute to the metastasis of melanoma by facilitating communication between melanoma cells and their surrounding endothelial cells [23]. Differential connexin expressions have been reported during melanocytic tumor progression, with in silico data showing downregulation of GJB2 mRNA [24]. In summary, studies support the functional role of CLDN10 and GJB2 in melanoma. GJB2 (Cx26) belongs to the connexin family, which comprises important components in gap junction-mediated intercellular communication [25]. As CLDN10 is a tight junction protein and Cx26 is a connexin, both represent possible candidates for TSGs in skin cancer [26]. We verify our data mining TSG candidates using wet-lab approaches such as DNA bisulfite conversion (BS), qualitative combined bisulfite restriction analysis (CoBRA), and pyrosequencing for quantifying methylation of specific CpGs in the chosen CGI. In addition to identifying TSG, the pipeline also includes guidance for designing methylation-specific primers for BS-converted DNA. Additionally, we focus on the relevance of isoform-specific TSG identification, which is often underestimated in online expression tools.

## 2. Results

### 2.1. Identification Strategy for Tumor Suppressor Genes

**Figure 1:**
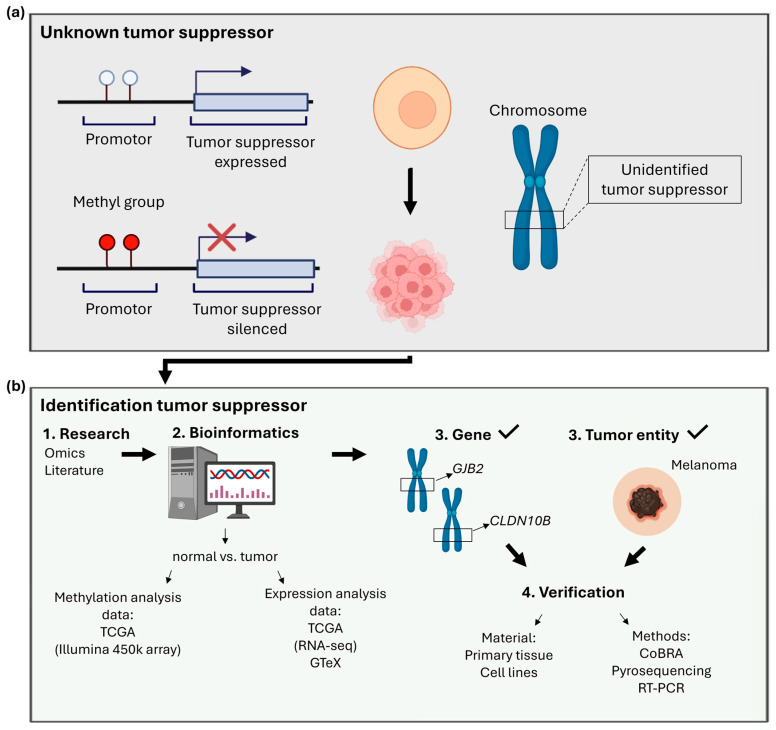
Graphical representation of the process for identifying hypermethylated tumor suppressors in cancer. (**a**) Unknown tumor suppressors that have been silenced by hypermethylation (**b**) can be identified using various bioinformatic online tools and datasets. Genes and the most relevant tumor entities can be verified in the wet-lab by pyrosequencing, combined bisulfite restriction analysis (CoBRA) and real-time (RT)-PCR of primary tissue and cell lines. Arrows and numbering indicate the workflow (Biorender: https://www.biorender.com).

Tumor suppressor genes (TSGs) are frequently silenced and functionally inactivated in cancer through epigenetic mechanisms, most notably DNA methylation. The identification of previously unrecognized TSGs therefore represents an important step toward a deeper understanding of tumor development and progression. With *CLDN10* and *GJB2* as representative examples in this study, we demonstrate an approach for discovering novel TSGs by combining systematic data mining using publicly available online tools with subsequent experimental validation in wet-lab assays. A common wet-lab biologist-friendly tool is the R2: Genomics Analysis and Visualization Platform, which allows analysis of expression and methylation datasets. R2 enables visualization of promoter-associated CpG methylation changes across tumor stages and diverse cancer entities. Gene expression levels in normal tissues and tumors were assessed using the GTEx dataset and GEPIA2, allowing the identification of cancer-associated expression changes potentially driven by epigenetic regulation. Promoter hypermethylation is frequently associated with transcriptional silencing [17]. However, this correlation is highly tumor entity-dependent, making tumor-specific analyses essential. Additional tools such as TCGA Wanderer and MethSurv were applied to refine promoter-specific CpG analyses and to integrate clinical outcome data, including patient survival. When consistent patterns of promoter hypermethylation, reduced gene expression, and unfavorable clinical outcome were observed in a specific tumor entity, bioinformatic findings were validated experimentally. For this purpose, we use the qualitative test of CoBRA and then quantify methylation by pyrosequencing primary tumors, metastases, and cancer cell lines of the same tumor entity. To quantify and correlate suppressed RNA transcription, we perform RT-PCRs. This approach to identifying a gene as an epigenetically silenced tumor suppressor is illustrated in Figure 1.

### 2.2. Bioinformatic Pipeline for Tumor Suppressor Gene Identification

**Figure 2:**
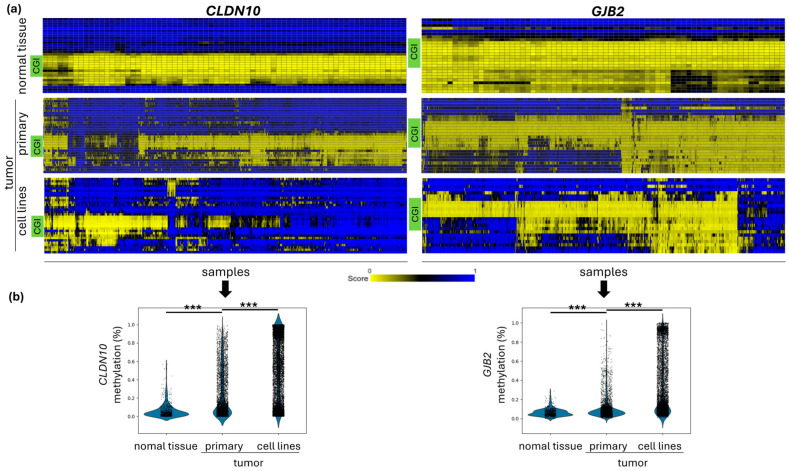
Increasing *CLDN10* and *GJB2* methylation with advanced tumor stage/progression. (**a**) DNA methylation status of CpG probes in the promoter region for *CLDN10* and *GJB2* in normal tissue, primary and cell line tumors (Illumina 450k array, Lokk, Heyn and Esteller datasets). The CpG island is shown in green (CGI) with hypomethylation in normal tissue (yellow) and increasing hypermethylation in primary tumors and tumor cell lines (blue) (Analysis R2, modified). (**b**) Increased methylation across *CLDN10B* and *GJB2* CGIs from normal to primary tumors and cancer cell lines is shown. Plots generated from the same Illumina 450k array data as mean CGI methylation for each sample. *** *p* < 0.001.

Methylation analysis by heat maps can be generated with the R2: Genomics Analysis and Visualization Platform for the selected example genes, such as *CLDN10* and *GJB2*. Both show an increased methylation with advancing tumor progression. An unmethylated promoter-associated CGI can be observed for both genes in various normal tissues. The corresponding CpG probes in the CGI show increased methylation with increasing stage from primary tumors to cell lines for *CLDN10* isoform B and *GJB2* (Figure 2a). The summary data illustrate the significant increase in methylation within the CGI from normal tissue to primary tumors and cancer cell lines (Figure 2b). From these results, the next step is to separate the methylation data by tissue type. This tissue-specific separation shows high methylation of the CGI of both genes in various entities, including melanoma cell lines (Figure S1). Using a ChIP-seq dataset, we observed a high transcriptional coverage of EZH2 and methylation-sensitive CCCTC-binding factor (CTCF) for both genes, supporting the possible epigenetic regulation (Figure S2).

**Figure 3:**
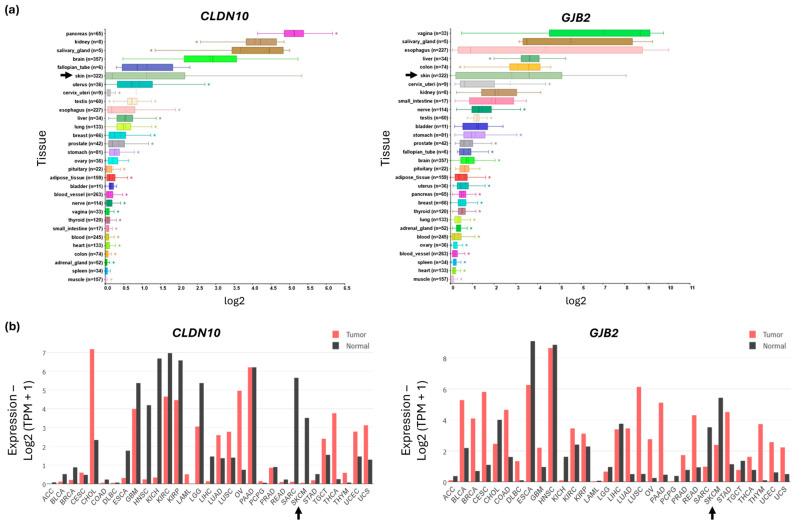
Expression levels of *CLDN10* and *GJB2* across normal and tumor tissue types. (**a**) *CLDN10* and *GJB2* expression in normal tissue. High endogenous expression level of *CLDN10* in the pancreas, kidney, salivary gland, brain, fallopian tube and skin. *GJB2* with high expression in the vagina, salivary gland, esophagus, liver, colon and skin. Skin is marked with an arrow (data GTEx v4, analyzed by R2). * *p* < 0.05. (**b**) Comparison of expression between tumor tissue (red) and normal tissue (black). Reduced median expression of *CLDN10* and *GJB2* in skin cancer (cutaneous melanoma; SKCM) as compared to normal skin tissue. SKCM is marked with an arrow (GEPIA2).

The GTEx v4 dataset was used to investigate *CLDN10* and *GJB2* expression in different tissue types and analyzed using R2: Genomics Analysis and Visualization Platform and GEPIA2. High *CLDN10* expression was found in normal tissue from the pancreas, kidney, salivary gland, brain, fallopian tube, and skin. High *GJB2* expression levels were found primarily in the vagina, salivary gland, esophagus, liver, colon, and skin (Figure 3a). It is also important to note the number of samples included in the datasets. While *CLDN10* expression in the salivary gland, kidney, or fallopian tube is evaluated using fewer than 10 samples, the brain and skin have several hundred samples. A similar observation can be made for *GJB2* expression, where the esophagus and skin are evaluated with high sample numbers.

Next, we investigated expression changes for both *CLDN10* and *GJB2*, comparing normal and tumor samples using GEPIA2. The analysis of normal and tumor tissue showed a significant decrease in expression in the Skin Cutaneous Melanoma (SKCM) data versus normal skin samples (Figure 3b).

**Figure 4:**
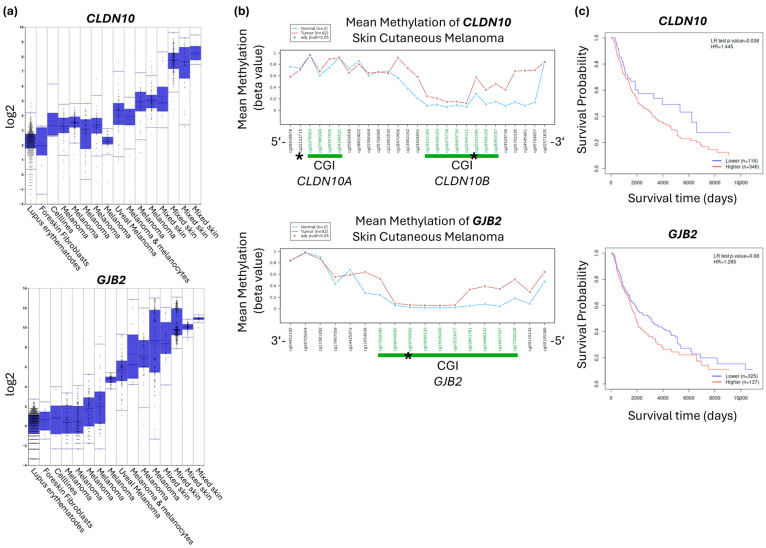
Reduced gene expression and hypermethylation of *CLDN10* and *GJB2* in skin cancer and gene hypermethylation correlates with reduced patient survival. (**a**) Expression levels of *CLDN10* and *GJB2* in various melanomas and normal skin tissue. The datasets show lower expression in melanomas compared to normal tissue. *CLDN10 p* = 1.01 × 10^−234^ (ANOVA). *GJB2 p* = 3.99 × 10^−247^ (ANOVA) (analyzed with R2). (**b**) Mean methylation of *CLDN10* and *GJB2* probes in melanomas compared to normal skin (analysis with Wanderer). The data indicates higher methylation of the probes in the CGI in melanomas (red) compared to normal skin (blue). The CGI is marked with a green bar. Regarding *CLDN10*, hypermethylation occurred only in the CGI of the isoform B; 5′ and 3′ show the orientation of the transcript. The TSS is marked with an asterisk (*). (**c**) Kaplan–Meier curves show a lower survival probability with high methylation of *CLDN10B* and *GJB2* in melanomas (Illumina 450 k Array, TCGA, Tumor Skin Cutaneous Melanoma (SKCM), analyzed by MethSurv).

With the increased methylation pattern across the CGIs and the low expression of *CLDN10* and *GJB2* in melanoma (Figure 2 and Figure 3), we next examined these two genes in the cancer type of malignant melanoma in more detail. Gene expression of *CLDN10* and *GJB2* from 16 different datasets of normal tissue and melanoma shows a lower expression level of both genes in melanoma compared to normal tissue (Figure 4a). The Wanderer tool is used to display individual CpG probes and their mean methylation in normal tissue and melanoma. CpG probes marked in green are in the CGI of the respective genes and show increased methylation in melanoma (Figure 4b). The representation of the individual CpGs also illustrates the relevance of isoform specificity and the probe position relative to the transcription start site. Two CGIs are annotated in green for *CLDN10*. Notably, the CGI of the *CLDN10A* isoform (cg20278383–cg04246521) does not show any altered methylation pattern in melanoma compared to normal tissue. However, there is a significant hypermethylation in the CGI of the *CLDN10B* isoform (cg16232183–cg18393747) (Figure 4b). Regarding the gene *GJB2*, however, the first four CpG probes of the CGI (cg02861781–cg27326226) in the 5′-3′ direction show an increased methylation pattern in melanoma, which is no longer evident in downstream CpGs of the CGI (Figure 4b). The selection of a suitable CpG probe for each gene was used to correlate CpG methylation with patient survival rates by Kaplan–Meier analysis. Data on malignant melanoma with high methylation of *CLDN10B* and *GJB2* showed a lower patient survival rate (Figure 4c).

### 2.3. Primer Design for Promoter Methylation Analysis

**Figure 5:**
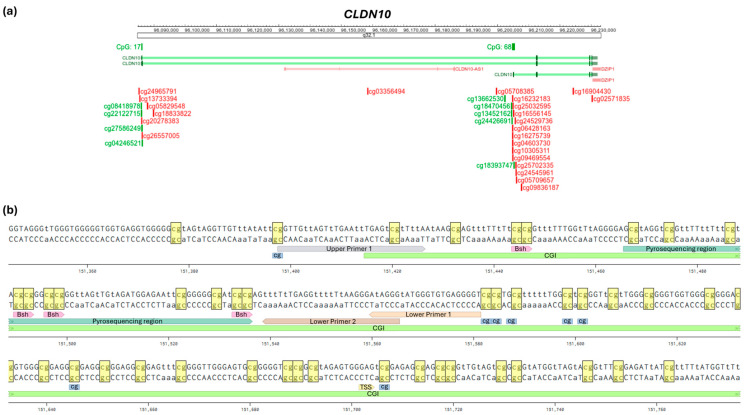
Genomic overview and primer design for *CLDN10* and wet-lab verification. (**a**) *CLDN10* gene overview with isoforms, CGIs (green bars, CpG: 68 for isoform A, CpG: 17 for isoform B) and cg-probes (R2 Genome Browser hg19). Green transcripts and probes located forward; red marks reverse. (**b**) BS-converted *CLDN10B* DNA sequence used for optimal primer design. The relevant region of the *CLDN10B* BS-converted sequence is shown. Semi-nested PCR-Product used for pyrosequencing analysis (length: 172 bp, Upper Primer 1, Lower Primer 1). Bsh = *Bsh1236I* cleavage site. cg = CpG probe. TSS = transcription start. Yellow = individual CpGs highlighted (Benchling, modified).

To verify the obtained bioinformatic results, it is essential to perform wet-lab-based validation of methylation levels. Therefore, we extracted the genomic DNA sequence of *CLDN10B* and *GJB2*. The DNA sequence was BS-converted and in silico and can be depicted through tools such as Benchling. Using this BS-DNA sequence, the CGI region to be analyzed was selected, with TSS and TLS marked; CpG sites are depicted with probes to enable correct PCR primer design. With the help of the R2: Genomics Analysis and Visualization Platform, the gene of interest can also be displayed with its exons, transcription and translation start sites, CGI position, and annotated CpG probes. The genomic overviews of *CLDN10* and *GJB2* can be seen in Figure 5a and Figure S3a. We use wet-lab analyses, such as CoBRA and pyrosequencing, both BS conversion-based, to verify the bioinformatic methylation data of our potential tumor suppressors.

Corresponding methylation analysis PCRs are performed with BS-treated DNA as a nested or semi-nested PCR. The overview of the *CLDN10B* gene is shown to illustrate the selection of the analyzed region (Figure 5b). The following guidelines were used: a region within the CGI +/− 400 bp away from the TSS, a PCR product of 120 bp to 350 bp with a melting temperature per primer between 53 °C and 62 °C (similar temperatures per primer pair, preferably with GC clamp), a CpG-rich region containing restriction sites for CoBRA, and an optimally overlapping pyrosequencing section. Primers were designed to pair in regions containing as few internal CpG sites as possible. For *GJB2*, the analyzed region was selected according to the same criteria (Figure S3b).

### 2.4. Lab-Based Verification of TSG Methylation and Expression

**Figure 6:**
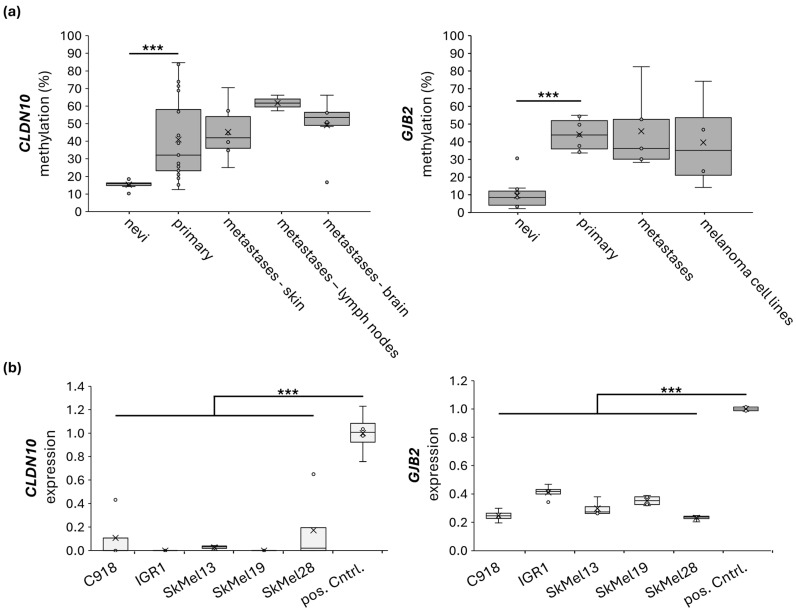
*CLDN10B* and *GJB2* are epigenetically silenced in melanoma samples through hypermethylation. Dots within the plots mark the individual samples; x marks the mean value. *** *p* < 0.001. (**a**) Pyrosequencing shows an increase in CLDN10B methylation with advancing tumor stage of melanoma (nevi *n* = 7, primary melanomas *n* = 21, metastases skin *n* = 6, metastases lymph node *n* = 2, metastases brain *n* = 6). The pyrosequenced GJB2 region shows an increase in methylation from nevi to primary melanomas (nevi *n* = 11, primary melanomas *n* = 7, metastases *n* = 5, melanoma cell lines *n* = 4). (**b**) Quantitative expression analysis in various melanoma cell lines (*n* = 5) shows lower CLDN10 and GJB2 expression. pos. Cntrl. = positive Control.

Using optimized PCRs, we performed CoBRA and pyrosequencing of various melanoma samples. For *CLDN10B*, we examined two different regions of its CGI (Figure 5b and Figure S3a). The CoBRA analysis showed increasing methylation with advancing tumor progression. For *CLDN10B*, 23% of nevus cell nevi (nevi), 68% of primary melanomas, 82% of metastases, and 100% of melanoma cell lines were hypermethylated (Figure S4). A similar pattern was observed for *GJB2*. Thirty-six percent of nevi, 86% of primary melanomas, 100% of metastases, and melanoma cell lines were hypermethylated (Figure S5). CoBRA covered three relevant CpGs for *CLDN10B* using the restriction enzyme *TaqI* and three relevant CpGs for *GJB2* using the enzyme *Bsh1236I* (Figure S3). We quantified CoBRA data using pyrosequencing. *CLDN10B* pyrosequencing showed a significant increase in methylation with increasing tumor stage. The nevi samples showed a mean methylation of 15%, the primary melanomas 44%, the skin metastases 45%, the lymph node metastases 62%, and the brain metastases 49% for *CLDN10B* (Figure 6a). *GJB2* pyrosequencing showed a significant increase in methylation from nevi to primary melanomas. The nevi samples examined showed an average methylation of 10%, primary melanomas 44%, metastases 46%, and melanoma cell lines 40% (Figure 6a). RNA expression analysis of melanoma cell lines showed a significant decrease in *CLDN10* and *GJB2* expressions compared to the control (Figure 6b).

## 3. Discussion

Epigenetic silencing of tumor suppressor genes (TSGs) by aberrant DNA methylation is a well-established hallmark of cancer [27]. In this study, we present a systematic and reproducible pipeline that combines bioinformatic screening with targeted experimental validation to identify epigenetically inactivated tumor suppressor genes. Applying this approach to malignant melanoma, we demonstrate that *CLDN10B* and *GJB2* exhibit promoter-associated CpG island hypermethylation accompanied by reduced gene expression and unfavorable clinical outcome, supporting their classification as epigenetically regulated tumor suppressors in this tumor entity.

A major strength of the presented pipeline is the stepwise refinement from publicly available large-scale datasets to gene-, isoform-, and CpG-specific experimental validation. Integrated analyses of TCGA- and GTEx-based platforms consistently revealed increasing promoter methylation in parallel with decreased expression during melanoma progression. Survival analyses further underscored the potential clinical relevance, as higher methylation levels of *CLDN10B* and *GJB2* were associated with reduced patient survival. The strong tumor entity specificity of DNA methylation patterns emerged as a central finding.

Aberrant promoter hypermethylation represents a common epigenetic mechanism and frequently affects TSGs involved in cell adhesion, differentiation, and intercellular communication [28,29,30,31]. In line with this concept, members of tight junction and gap junction gene families are recurrent targets of epigenetic inactivation in cancer [32,33,34,35,36]. CLDN10, a component of tight junctions, is implicated in maintaining epithelial barrier function and cellular polarity, both of which are frequently disrupted during tumor invasion and metastasis [37,38]. Similarly, *GJB2*, encoding Connexin 26, plays a critical role in gap junction-mediated intercellular communication, a process often lost during malignant transformation [39,40]. Epigenetic silencing of these genes may therefore contribute to increased cellular plasticity, invasiveness, and metastatic potential in melanoma displaying an epithelial–mesenchymal transition (EMT) phenotype [23,41,42,43,44]. Although *CLDN10* and *GJB2* are expressed in various normal tissues, their epigenetic repression was particularly pronounced in melanoma. Consistent with our findings in melanoma, epigenetic regulation of *CLDN10* and *GJB2* has also been reported in other malignancies. We have already reported hypermethylation of *CLDN10B* in renal cancer, consistent with the study by Yang et al. [20,21]. In addition, reduced *CLDN10* expression has been described in gastric, breast and lung cancer, suggesting a broader role for CLDN10 as a tumor suppressor [45,46,47]. Similarly, *GJB2* has been reported to undergo promoter hypermethylation or aberrant expression in several epithelial cancers, including colorectal and gastric cancer [36,48,49]. *CLDN10B* and *GJB2* appear to be epigenetically silenced tumor suppressors via promoter hypermethylation in melanoma. Therefore, one could argue that DNA-demethylating epi-drugs might restore their expression. However, agents such as decitabine act systemically and induce genome-wide hypomethylation rather than selectively reactivating only the target gene, raising concerns about broad off-target effects [50]. Epi-drugs such as decitabine are FDA- and EMA-approved treatments for non-solid tumors such as myelodysplastic syndromes, a type of leukemia [51]. The DNA methyltransferase inhibitor is a cytidine analog, which induces passive demethylation with progressive replication [52]. This underscores that it is not a melanoma-specific or gene-targeted therapy. It has been reviewed that, despite well-demonstrated activity in myeloid malignancies, decitabine has shown limited utility in solid tumors [53]. The therapeutic potential of both claudins and connexins is being discussed, including drug response, for example, to antibody therapy in cancer [54,55]. Due to the loss of expression in cancer, a classical antibody or inhibitor therapy blocking the function of oncogenes is not possible. However, we hypothesize that stabilizing the proteins themselves could increase residual CLDN10 and GJB2 levels and may represent a therapeutic approach.

Accordingly, our pipeline emphasizes early entity-focused analyses to distinguish epigenetic inactivation from alternative regulatory mechanisms. Importantly, we observed a clear isoform-specific methylation pattern for *CLDN10*, with hypermethylation restricted to the CpG island associated with the *CLDN10B* isoform, while the *CLDN10A*-associated CpG island remained largely unmethylated. Similar *CLDN10* isoform-specific differences have already been observed in renal clear cell carcinoma [21]. It is important to note that most online expression analysis tools do not distinguish between isoforms and instead report mean gene expression levels, which can mask the expression of the isoform of interest. Therefore, a more in-depth analysis is required, involving bioinformatic analysis of RNA-seq data at the exon level using sequencing reads. In summary, this underscores the necessity of isoform-aware annotation during bioinformatic screening and primer design, as gene-level analyses may overlook biologically relevant epigenetic alterations.

Experimental validation using CoBRA and pyrosequencing confirmed the bioinformatic predictions and demonstrated a progressive increase in methylation from benign nevi to advanced melanoma stages across primary tumors, metastases, and cell lines. The combination of fast and cost-effective restriction-based screening and single-nucleotide level resolution by quantitative pyrosequencing proved to be an efficient and robust strategy for validating candidate tumor suppressors in clinical material. Importantly, CoBRA and pyrosequencing are well suited for the analysis of clinical material, as both methods can be applied to fixed and archived samples. Moderate DNA degradation has only a limited impact on these bisulfite-based, PCR-driven approaches. In contrast, array-based technologies are strongly affected by DNA degradation. Consequently, CoBRA and pyrosequencing enable reliable methylation analysis of retrospective clinical material. Several limitations in the present study should be considered. Sample numbers for certain metastatic subgroups were limited, and functional assays were beyond the scope of this study. The next steps in experimental validation may include pharmacological methylation inhibition via 5-Aza-2′deoxycytidine (decitabine). Additionally, functional studies to reactivate the genes of interest, including gene overexpression experiments, could be performed in melanoma cell lines. Moreover, the pipeline presented here focuses on DNA methylation and does not study additional epigenetic mechanisms such as histone modifications [56,57,58]. Notably, the reproducibility of our computational prioritization strategy has been supported by our prior melanoma study identifying RIPK3 as a tumor suppressor silenced by hypermethylation, providing an established proof-of-principle for the framework applied here [6].

In summary, we present a practical and versatile pipeline for identifying epigenetically inactivated tumor suppressor genes, particularly suited for wet-lab-oriented research groups. Using *CLDN10* and *GJB2* as examples in skin cancer of malignant melanoma, we demonstrate how integrative bioinformatic analyses combined with targeted methylation assays can reveal tumor- and isoform-specific epigenetic alterations with potential biological and clinical relevance.

## 4. Materials and Methods

### 4.1. Online Tools

R2: Genomics Analysis and Visualization Platform (R2) (http://r2.amc.nl (accessed on 3 March 2026)). A single-dataset analysis was used to generate the Illumina array-based DNA methylation heat maps. Selected datasets, specified as GEO IDs, were 450 k arrays: GSE50192, GSE76269, and GSE68379 (Supplementary Materials Table S1). Methylation data analysis was performed with the “view gene” setting and ‘View all Met_ids for a gene (Heatmap)’. To proceed, the gene name was entered. The gene methylation overview created in this process was used to select relevant regions such as CGIs. The depiction can be further sorted by tissue type through ‘Order samples by track’. Regarding expression analysis, a single-dataset analysis was used as well to display expression data from normal tissue regarding the expression of *CLDN10* and *GJB2*—selected dataset: ensgtexv4 (GTEx v4). Using the ‘clinisnitch’ option, the box plot sorted by tissue can be generated if there is a sufficient and significant correlation of expression with tissue type. An across-dataset analysis was used to compare various skin expression datasets for *CLDN10* and *GJB2* expression. Chip type u133p2 and normalization scheme MAS5.0 were used (hs, u133p2, MAS5.0). Selected datasets were GSE4217, GSE35389, GSE31534, E-MTAB-3296, GSE7127, GSE10916, GSE33643, GSE22138, GSE130244, GSE19234, GSE7553, GSE4587, GSE13355, GSE65127, and GSE45512. All datasets used from R2 are listed in Table S1.

GEPIA 2 (http://gepia.cancer-pku.cn (accessed on 3 March 2026)). Using the gene IDs CLDN10 and GJB2, bar plots with gene expression profiles across all tumor samples vs. normal tissue were generated. Datasets: Tumor (TCGA), Normal (TCGA and GTEx).

TCGA Wanderer (http://maplab.imppc.org/wanderer/ (accessed on 3 March 2026)) [59]. TCGA data was used to analyze individual CpG probes and their mean methylation in normal tissue and melanoma. With the corresponding gene ID, the TCGA Skin Cutaneous Melanoma (SKCM) dataset from a 450 k methylation array was selected as the data type. The *p*-value threshold was set at *p* < 0.05.

MethSurv (https://biit.cs.ut.ee/methsurv/ (accessed on 3 March 2026)) [60]. This was used to generate Kaplan–Meier survival curves for *CLDN10* and *GJB2* in skin cancer—dataset: Skin Cutaneous Melanoma (SKCM) TCGA. The analyzed probe for *CLDN10* is in the selected region up to 200 bp upstream of its transcription start site (TSS). The analyzed probe for *GJB2* is in the selected region up to 1500 bp upstream of its TSS. *CLDN10*: TSS200 (−118 bp), cg06428163. *GJB2*: TSS1500 (−702 bp), cg27326226.

### 4.2. Wet-Lab Methylation and Expression Analyses

DNA from melanoma cell lines was isolated by stepwise phenol-chloroform-isoamyl alcohol extraction (Carl Roth GmbH, Karlsruhe, Germany). DNA from primary tissue was isolated by the NucleoSpin Tissue Kit (MACHEREY-NAGEL 740,952.250, Aachen, Germany). Promoter methylation was analyzed by combining bisulfite restriction analysis (CoBRA) and bisulfite pyrosequencing. For bisulfite treatment, we used the EZ DNA Methylation Kit (Zymo Research D5001, Zymo Research Europe GmbH, Freiburg, Germany). To perform CoBRA and pyrosequencing, bisulfite-treated DNA was PCR-amplified (with 0.2 mM dNTP mix, 1.5 mM MgCl_2_, 10 pmol of each primer, 1.5 U Taq polymerase, 50 cycles). Benchling was used for primer design (https://www.benchling.com (accessed on 3 March 2026)). *CLDN10B* was amplified with a semi-nested PCR, and *GJB2* was amplified with a nested PCR. PCR conditions must be adapted to the target gene and primer set used. The enzymes *TaqI* or *Bsh1236I* (Thermo Fisher Scientific, Dreieich, Germany) were used for the restriction digestion of CoBRA. The *TaqI* restriction site is 5′-TCGA-3′, and the *Bsh1236I* restriction site is 5′-CGCG-3′. For digestion, 5 µL of PCR product (prior control in 2% agarose gel is recommended) was incubated with 1 U/µL enzyme for one hour at 65 °C or 37 °C (*TaqI* or *Bsh1236I*, respectively). Gene-specific methylation quantification of primary samples was performed by pyrosequencing with PyroMark Q24 (QIAGEN GmbH, Hilden, Germany). Eleven CpGs are included in the *CLDN10B* analyzed region, and eight CpGs are included in the *GJB2* analyzed region. RNA was isolated using Isol-RNA lysis procedure (Trizol, Thermo Fisher Scientific, Dreieich, Germany). For RNA expression analysis, quantitative real-time PCR was performed with SYBR Select (Thermo Fisher Scientific, Dreieich, Germany) using Rotor-Gene 3000 (QIAGEN GmbH, Hilden, Germany). The cell lines were normalized to *GAPDH* and analyzed in technical quadruplicate. PCR primers and sequencing primers that were used to analyze promoter methylation and RNA expression are listed in Table S2.

### 4.3. Statistical Analysis

*p*-values: * *p* < 0.05, ** *p* < 0.01, *** *p* < 0.001. On plots generated from the same Illumina 450 k array data, a Mann–Whitney U rank test (SciPy v1.14.1, Python v3.12.5) was performed. Bonferroni-adjusted *p*-values indicated statistically significant differences for all pairwise comparisons. Pyrosequencing and RT-PCR: Microsoft Excel was utilized for statistical analysis. A one-tailed Welch’s *t*-test was applied to distinguish between two groups of samples.

### 4.4. Tissue and Cell Lines

The melanoma cell lines C918 (RRID: CVCL_8471), IGR1 (RRID: CVCL_1303), SkMel13 (RRID: CVCL_6022), SkMel19 (RRID: CVCL_6025) and SkMel28 (RRID: CVCL_0526) were used. The cell lines HeLa (RRID: CVCL_0030) and HEK293T (RRID: CVCL_0063) served as methylation controls. All cell lines were mycoplasma-free and authenticated using short tandem repeat (STR) profiling within the last 3 years (Eurofins Genomics, Ebersberg, Germany). All 82 primary samples used are listed in the Supplementary Materials (Supplementary Materials Table S3). The study was conducted according to the Declaration of Helsinki Principles. All patients signed informed consent at the initial clinical investigation. The study was approved by local ethics committees [21,35].

## Data Availability

Although no new omics data has been generated, researchers are willing to provide additional information on protocols and samples upon request.

## Supplementary Materials

The following supporting information can be downloaded at https://www.mdpi.com/article/10.3390/ijms27052483/s1.
