# Supplementary material for: A Bioinformatics and Wet-Lab-Based Pipeline Identifies CLDN10 and GJB2 as Epigenetically Silenced Tumor Suppressor Genes in Cutaneous Melanoma
Source: Int J Mol Sci. 2026 Mar 8;27(5):2483. doi: 10.3390/ijms27052483 (PMC12986398; doi:10.3390/ijms27052483)
Supplement: Supplementary file 1 [file ijms-27-02483-s001.zip › ijms-4174922-supplementary.pdf]

Supplementary material

Table S1: All data sets used from R2.

R2 dataset	(GEO) ID	Used for
Normal Tissues - Lokk - 70 - custom - ilmnhm450<br><br>Coronary artery, splenic artery, thoracic aorta, abdominal aorta, urinary bladder, articular cartilage, bone, subcutaneous and abdominal adipose tissue, lymph node, gastric mucosa, gallbladder, sciatic nerve, medulla oblongata, red and yellow bone marrow, tonsils	GSE50192	Methylation Heatmaps
Tumor Types (landscape) - Heyn - 493 - custom - ilmnhm450	GSE76269	
Cell line Cancer Pharmacogenomic - Esteller - 1028 - custom - ilmnhm450	GSE68379	
Normal Tissues GTeX v4 - GTeX - 2921 - RPKM - ensgtexv4<br><br>vagina, salivary gland, esophagus, liver, colon, skin, cervix (uterus), kidney, small intestine, nerve, testis, bladder, stomach, prostate, fallopian tube, brain, pituitary, adipose tissue, uterus, pancreas, breast, thyroid, lung, adrenal gland, blood, ovary, blood vessel, spleen, heart muscle	ensgtexv4	Expression normal tissue
Disease Lupus erythematosus (skin) - Tee - 704 - MAS5.0 - u133p2	gse185047	Expression skin
Exp Foreskin Fibroblasts Spheroid Formation - Jack - 12 - MAS5.0 - u133p2	GSE4217	
Exp Cellline Melanoma-Exosome - McMasters - 8 - MAS5.0 - u133p2	GSE35389	
Exp Melanoma A375 siRNA - Wang - 51 - MAS5.0 - u133p2	GSE31534	
Mixed Melanoma Immunotherapy (HIV infection) - vandenHam - 90 - MAS5.0 - u133p2	E-MTAB-3296	
Cell line Melanoma - Packer - 63 - MAS5.0 - u133p2	GSE7127	
Exp Melanoma - Augustine - 50 - MAS5.0 - u133p2	GSE10916	
Cell line Melanoma pi3K-BKM120 - Gaulis - 40 - MAS5.0 - u133p2	GSE33643	
Tumor Uveal Melanoma - Saule - 63 - MAS5.0 - u133p2	GSE22138	
Exp melanoma and melanocyte CD271 - Filipp - 12 - MAS5.0 - u133p2	GSE130244	
Tumor Melanoma Metastatic - Bhardwaj - 44 - MAS5.0 - u133p2	GSE19234	
Tumor Melanoma (Metastatic) - Matta - 87 - MAS5.0 - u133p2	GSE7553	
Mixed Skin (Melanoma) - Becker - 18 - MAS5.0 - u133p2	GSE4587	
Mixed Skin Psoriatic - Nair - 180 - MAS5.0 - u133p2	GSE13355	
Mixed Skin Vitiligo - Passeron - 40 - MAS5.0 - u133p2	GSE65127	
Mixed Skin Alopecia Areata - Xing - 10 - MAS5.0 - u133p2	GSE45512	

Table S2: PCR primers and sequencing primers for methylation analysis.

Gene	Primer	Sequence 5'-3'
CLDN10	Primer U1	GGTTGTTAGTTTGAATTTGAGTYGTTTTA
CLDN10	Primer L1	CCCTATCCCTTAAAAAACCTCAAAAAA
CLDN10	Primer L2	ACCCCTCACACCCATACCCTAT
CLDN10	Pyrosequence Primer	GGTTTTTTGGTTTAGGGGA
CLDN10	CoBRA Primer U1	TTTATTTTAGGTTGGGTATTGGTGTTT
CLDN10	CoBRA Primer L1	CCAACATAAAAAAAAAATCCTTACAATTAA
CLDN10	RT-PCR Primer U1	ACCGACTACTGGAAGGTGTC
CLDN10	RT-PCR Primer L1	GTATATAACCGTCCAGCGCCA
GJB2	Primer U1	GTTAAG GGG TTGGGGGAGG
GJB2	Primer L1	CCTTTTAACRGCACCCCACACCC
GJB2	Primer U2	GGAGATTTAGGGYGTTGGGGG T
GJB2	Primer L2	ACCCRGCCTCTTCCCTRGAAAACT
GJB2	Pyrosequence Primer	GAAGGGGATTTGGGGGGTT
GJB2	RT-PCR Primer U1	CTCCCGACGCAGAGCAAA
GJB2	RT-PCR Primer L1	GTAGCACACGTTCTTGCAGC
GAPDH	RT-PCR Primer U1	TGGAGAAGGCTGGGGCTCAT
GAPDH	RT-PCR Primer L1	GACCTTGGCCAGGGGTGCTA

Table S3: Primary samples used for pyrosequencing.

Sample ID	Tissue	Sample ID	Tissue	Sample ID	Tissue
NZN8		MM9		MM22	
NZN13		MM10		MM60	
NZN15		MM12		MM61	
NZN16		MM26		MM78	
NZN17	nevi	MM34	primary tumor	MM80	metastases skin
NZN18		MM36		MM81	
NZN19		MM39		MM83	
NZN23		MM40		MM86	
NZN24		MM41		MM87	

NZN26		MM42		MM111	
NZN27		MM43		MM121	
NZN30		MM44		MM122	
NZN31		MM47		MM15	
NZN33		MM49		MM59a	
NZN34		MM51		MM118	metastases lymph node
NZN37		MM53		MM120	
NZN42		MM63		MM153	
NZN46		MM65		MM157	
NZN50		MM67		NP1	
NZN51		MM68		NP3	
NZN53		MM69		NP4	
NZN54		MM70		NP5	
NZN57		MM72		NP6	
		MM74		NP8	metastases brain
		MM100		NP9	
		MM101		NP18	
		MM104		NP20	
		MM107			
		MM109			
		MM127			
		MM137			
		MM138			

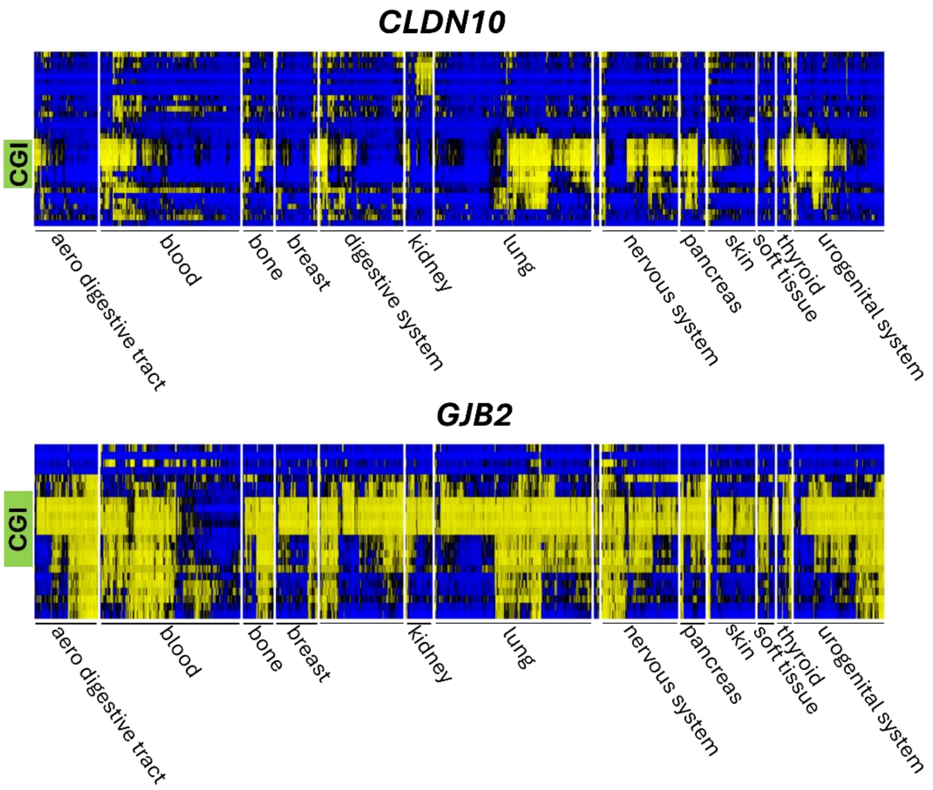

Figure S1: DNA-Methylation status of *CLDN10* and *GJB2* in cancer cell lines sorted by tissue. The CpG island is shown in green (CGI) with hypomethylation in yellow and hypermethylation in blue. (Ilumina 450k array, Esteller datasets, analysis R2, modified).

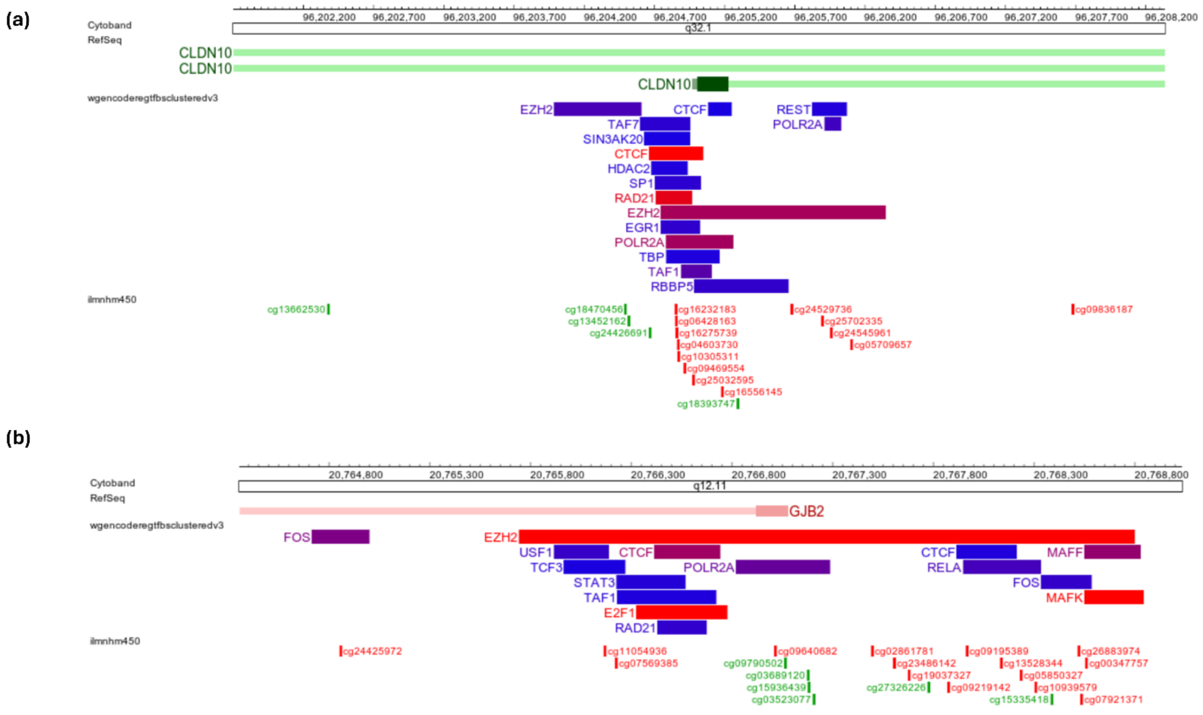

Figure S2: *CLDN10 and GJB2* with transcriptional regulators and CpG probes. (a) *CLDN10* gene overview and the transcriptional regulators such as EZH2 or CTCF binding at the promoter region. **(b)**

GJB2 gene overview and the transcriptional regulators. Green transcripts and probes located forward, red marks reverse located (ChIP-seq in ENCODE by R2).

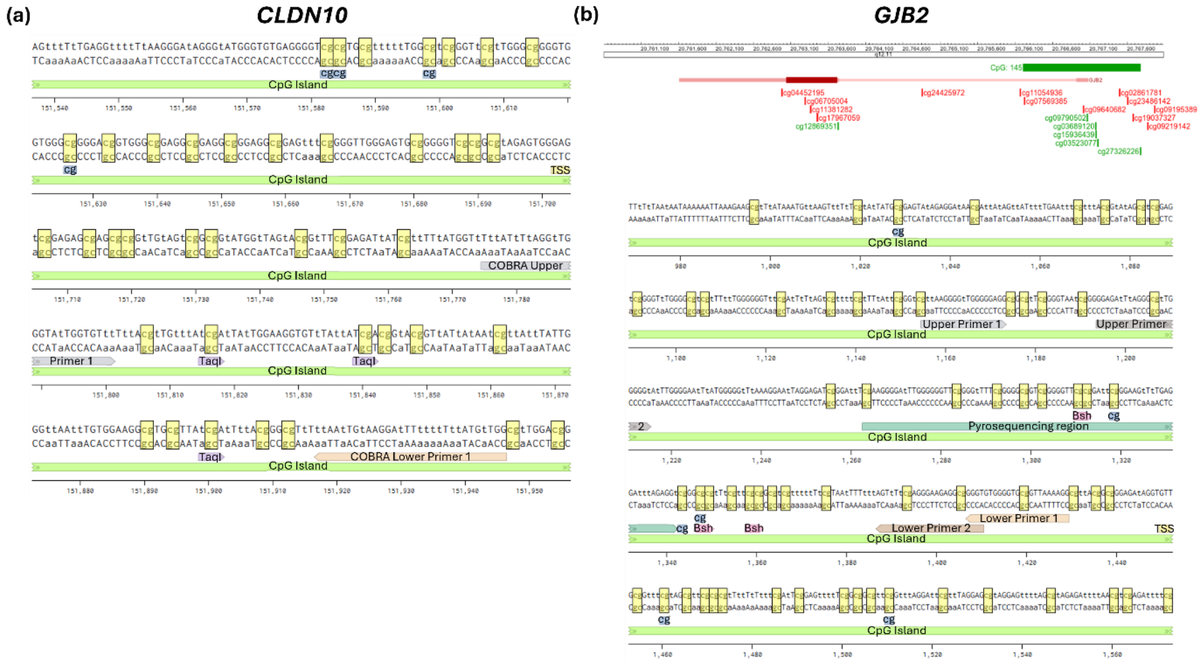

Figure S3: CoBRA primer design and genomic overview for *CLDN10* and *GJB2*. Bsh=*Bsh1236I* cleavage site. TaqI=*TaqI* cleavage site. cg =CpG probe. TSS=transcription start. Yellow = individual CpGs highlighted. **(a)** Bisulfite-converted *CLDN10* DNA sequence used for optimal primer design. PCR-Product used for CoBRA analysis (length: 172bp, Upper Primer 1, Lower Primer 1). **(b)** *GJB2* gene owerview, CpG-Island (green bar, CpG: 145) and cg-probes (R2 Genome Browser hg19). Green transcripts and probes located forward, red marks reverse located. Bisulfite-converted *GJB2* DNA sequence used for optimal primer design is shown below. Fully-nested PCR-Product used for pycosequencing and CoBRA analysis (length: 217bp, Upper Primer 2, Lower Primer 2). (Benchling, modified).

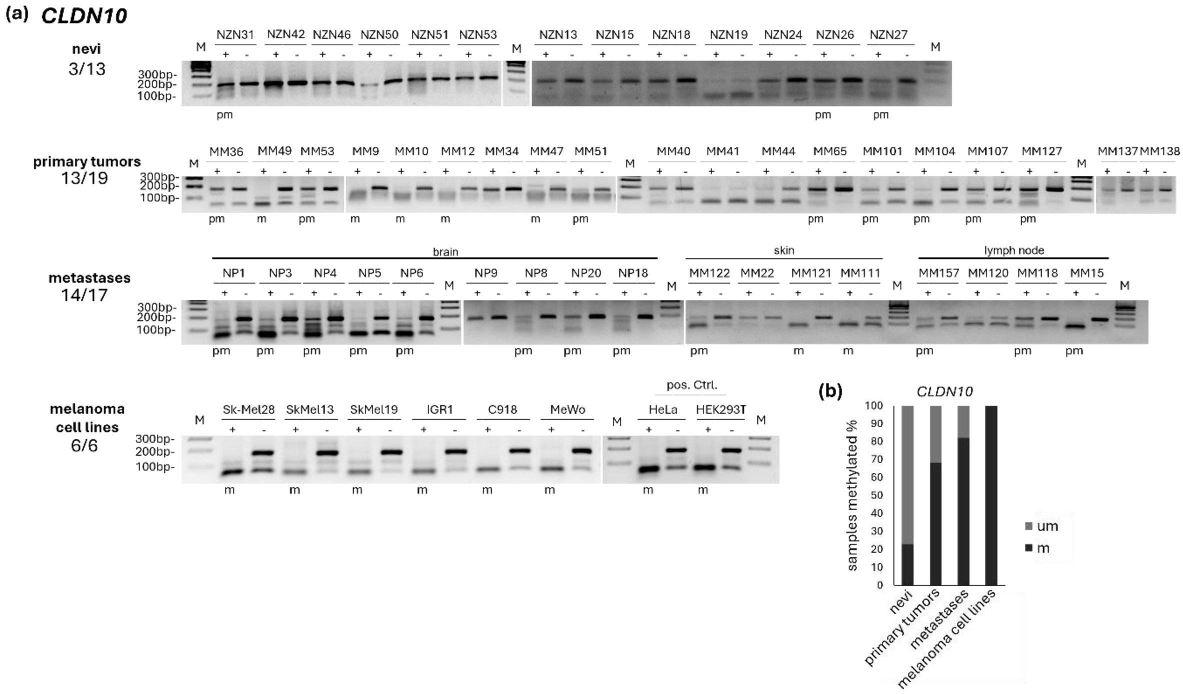

Figure S4: CoBRA methylation analysis shows increasing *CLDN10* methylation with advancing melanoma progression. (a) After digestion with *TaqI* (+), fragments of 42 bp, 24 bp, 60 bp, and 46 bp were expected when methylated. The uncut fragment has a length of 172 bp. A mock digestion without enzyme (-) served as a control. HeLa and HEK293T DNA, which represent complete digestion, were used as positive controls (pos. Ctrl.). The evaluation was performed using 2% agarose gel electrophoresis. bp = base pairs. M = 100 bp marker. pm = partially methylated, vm = fully methylated. **(b)** Summary of the CoBRA results from (a) Percentage of methylated (m) samples compared to unmethylated (um) samples.

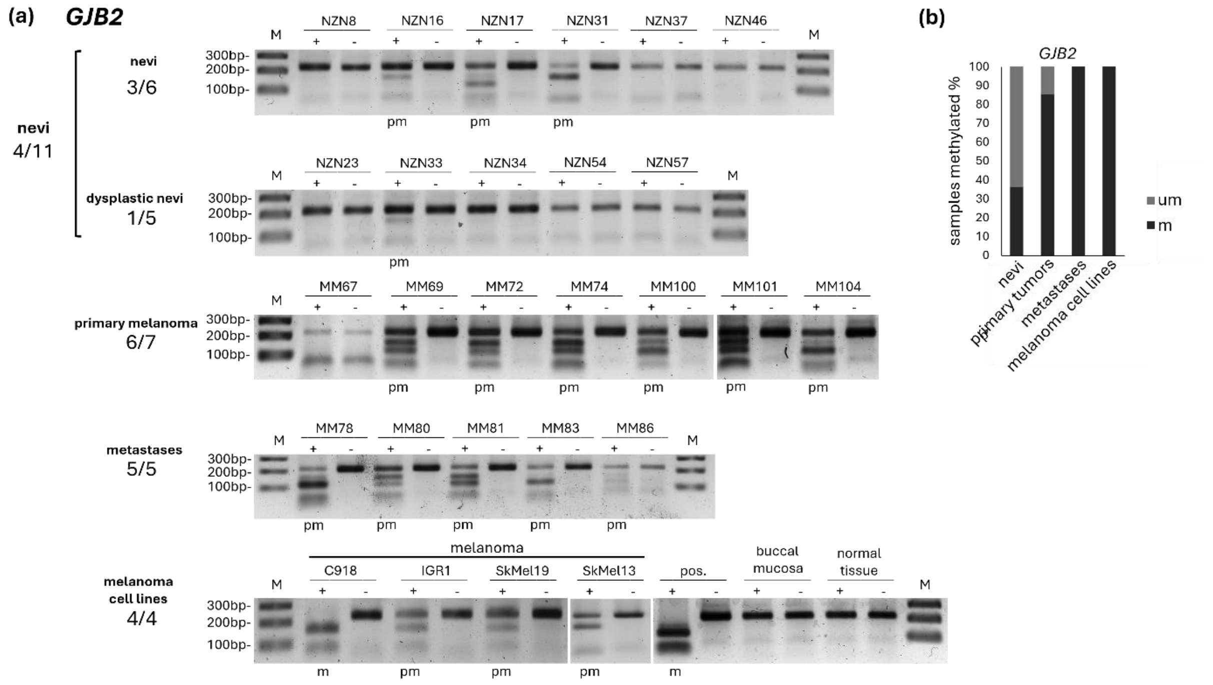

Figure S5: CoBRA methylation analysis shows increasing *GJB2* methylation with advancing melanoma progression. (a) After digestion with *Bsh1236I* (+), fragments of 118 bp, 37 bp, 11 bp, and 51 bp were expected when methylated. The uncut fragment has a length of 217 bp. A mock digestion without enzyme (-) served as a control. *In vitro* methylated DNA, which represents complete digestion, was used as a positive control (pos.). *Buccal mucosa* and normal skin tissue were used as negative controls. The evaluation was performed using 2% agarose gel electrophoresis. bp = base pairs. M = 100 bp marker. pm = partially methylated, vm = fully methylated. **(b)** Summary of the CoBRA results from a. Percentage of methylated (m) samples compared to unmethylated (um) samples.
